# (Sub)clinical cardiovascular disease is associated with increased bone loss and fracture risk; a systematic review of the association between cardiovascular disease and osteoporosis

**DOI:** 10.1186/ar3224

**Published:** 2011-01-17

**Authors:** Debby den Uyl, Mike T Nurmohamed, Lilian HD van Tuyl, Hennie G Raterman, Willem F Lems

**Affiliations:** 1 Department of Rheumatology, VU Medical Centre, De Boelelaan 1117, 1081 HV Amsterdam, The Netherlands; 2 Department of Internal Medicine, VU Medical Centre, De Boelelaan 1117, 1081 NV Amsterdam, The Netherlands; 3 Department of Rheumatology, Jan van Breemen Research Institute/Reade, Dr Jan van Breemenstraat 2, 1056 AB Amsterdam, The Netherlands

## Abstract

**Introduction:**

Both cardiovascular disease and osteoporosis are important causes of morbidity and mortality in the elderly. The co-occurrence of cardiovascular disease and osteoporosis prompted us to review the evidence of an association between cardiovascular (CV) disease and osteoporosis and potential shared common pathophysiological mechanisms.

**Methods:**

A systematic literature search (Medline, Pubmed and Embase) was conducted to identify all clinical studies that investigated the association between cardiovascular disease and osteoporosis. Relevant studies were screened for quality according to guidelines as proposed by the Dutch Cochrane Centre and evidence was summarized.

**Results:**

Seventy studies were included in this review. Due to a large heterogeneity in study population, design and outcome measures a formal meta-analysis was not possible. Six of the highest ranked studies (mean *n *= 2,000) showed that individuals with prevalent subclinical CV disease had higher risk for increased bone loss and fractures during follow-up compared to persons without CV disease (range of reported risk: hazard ratio (HR) 1.5; odds ratio (OR) 2.3 to 3.0). The largest study (*n *= 31,936) reported a more than four times higher risk in women and more than six times higher risk in men. There is moderate evidence that individuals with low bone mass had higher CV mortality rates and incident CV events than subjects with normal bone mass (risk rates 1.2 to 1.4). Although the shared common pathophysiological mechanisms are not fully elucidated, the most important factors that might explain this association appear to be, besides age, estrogen deficiency and inflammation.

**Conclusions:**

The current evidence indicates that individuals with prevalent subclinical CV disease are at increased risk for bone loss and subsequent fractures. Presently no firm conclusions can be drawn as to what extent low bone mineral density might be associated with increased cardiovascular risk.

## Introduction

Cardiovascular (CV) disease and osteoporosis are both important causes of morbidity and mortality in aging men and women. They share common risk factors, such as increased age and inactivity, and are frequently found in the same individuals, suggesting a possible relationship. Results from epidemiological studies indicate an association between CV disease and osteoporosis. Prevalent CV disease and subclinical atherosclerosis have been found to be related to low bone mass and increased fracture risk [1-4]. Similarly, low bone mineral density (BMD) has been related to increased cardiovascular risk [5-8]. This relationship is often regarded as a result of aging; however, recent evidence suggests a direct association, independent of age and traditional cardiovascular risk factors and accumulating evidence from experimental research indicates a shared pathogenesis. A variety of factors that influence bone metabolism are involved in the development of vascular disease, for example, atherosclerosis and vascular calcification. Interestingly, several bone-related proteins are implicated in the calcification process resulting in mineral deposition [9]. This is important as calcification of the arterial wall may be a marker for CV disease and was shown to predict CV events [10]. Given the importance of identifying a person at risk for CV events or fractures, evidence for an association of CV disease with osteoporosis might have implications for screening decisions in patients with low bone mass and vice versa. This review aims to summarize all the present clinical literature about the association between CV disease and osteoporosis and to describe common pathophysiological mechanisms. The results of this review are grouped into two topics: clinical results, discussing the relationship between 1) cardiovascular disease and osteoporosis and 2) vice versa. In addition, the possible pathophysiological links of CV disease and osteoporosis will be discussed.

## Materials and methods

### Search strategy

A systematic search (in Medline, Pubmed and Embase) was conducted to identify all clinical studies from 1966 to January 2010 (last updated 8 June 2010) that investigated the association between cardiovascular disease and osteoporosis. The following search terms for cardiovascular disease were used: cardiovascular diseases, cerebrovascular diseases and peripheral vascular diseases. These searches were each combined with an osteoporosis search block and duplicates were removed. Searches were limited to human studies in the English, Dutch and German languages. The complete Medline search is available in Additional file 1. In addition, references from the retrieved articles were scanned for additional relevant studies.

### Selection criteria

Abstracts were screened by one reviewer (DdU) and studies were included in the review if they fulfilled the following inclusion criteria: epidemiological studies (including prospective, cross-sectional, case-control, or retrospective studies) reporting the association between CV disease and osteoporosis in the general population or in patients with prevalent CV disease or low bone mass. Cardiovascular disease was defined as coronary heart disease (CHD) (myocardial infarction, angina pectoris, coronary insufficiency or ischemic heart disease), cerebrovascular disease (stroke, transient ischemic attacks), peripheral arterial disease (PAD) (lower extremity claudication, arterial thrombosis/embolism, ankle brachial index (ABI) <0.90) or subclinical atherosclerosis measured as intima media thickness (IMT) or vascular calcification. In addition, bone mass had to be assessed as bone mineral density or bone quality, and osteoporosis was defined as low bone mass (T-score ≤-2.5) or increased fracture risk (vertebral and non-vertebral). Exclusion criteria were: reviews, letters, case-reports, intervention studies and biomechanical studies. Studies in patients with co-morbidity other than osteoporosis or CV disease were also excluded. Finally, investigations using risk factors of CV disease or osteoporosis as outcome measurements, such as hypertension, metabolic syndrome, atrial fibrillation, bone markers, and calcium supplementation were not included.

### Assessment of study quality

The quality of each manuscript was systematically assessed with a checklist for cohort studies as proposed by the Dutch Cochrane Collaboration [11] (Additional file 2). Quality assessment included a scoring of the following components: definition of study population, the likelihood of bias, adequate blinding, the accuracy of outcome measurements, duration of follow-up and selective loss-to follow-up, the appropriateness of the statistical analysis and the clinical relevance. All items had the following answer options: yes/no/too little information to answer the question. We considered incomplete information or data important criteria for study quality. Therefore, if the answer could not be given because the study provided too little information, a negative score (for example, "no") was given. Each "no" was scored and an equal weight was given to each item. A maximum of 10 points could be given. The scores of each study are given in Tables 1 and 2.

**Table 1:** Prospective studies investigating relationship CV disease and low BMD

Study	Study population (years follow-up)	Number of cases (% women)	Postmenopausal women	CV disease excluded	Mean age	Outcome CV disease	Outcome bone mass	Results #	Quality
Sennerby, 2009 [13]	Population-based (20)	31,936 (NA)	NA	Yes	67.9 to 74.4	CV disease by National patient registry, ICD 9 codes	Incident hip fracture by National patient registry, ICD 9 codes	Women: HR: 4.42 (95% CI 3.49 to 5.61) Men: HR: 6.65 (95% CI 4.82 to 9.19)	3
Szulc, 2008 [14]	Population-based (10)	781 (0%)	No	No	65	AC by X-spine	Incident fracture by hospital records or X-ray	OR: 2.54 to 3.04 (*P *< 0.005 to 0.001)	3
Naves, 2008 [4]	Population-based (4)	624 (51%)	NA	No	65	AC by X-spine	BMD lumbar spine and femur by DXA Incident fracture by hospital record or X-ray	Change BMD spine in progression AC vs no progression AC: -1.48% vs 1.43% (*P *<.0001) Change BMD hip in progression AC and no progression AC: -0.48% vs 0.23% (*P *= 0.315) Incident fracture: OR: 2.13 (95% CI 0.85 to 5.31)	3
Von Muhlen, 2009 [15]	Population-based (4)	1,332 (60%)	NA	No	73.8	PAD by ABI	BMD lumbar spine and hip by DXA and incident fracture by X-ray	Women: Change BMD in PAD vs no PAD: 59.2% vs 43.5% (*P *< 0.05) Incident non-vert fracture: OR: 0.84 (95% CI 0.31 to 2.26) Men : Change BMD in PAD vs no PAD : 43.5% vs 35.5% (*P *= 0.20) Incident non-vert fracture: OR: 1.52 (95% CI 0.30 to 7.45)	3
Collins, 2009 [2]	Population-based (5.4)	4,302 (0%)	NA	No	73.5	PAD by ABI	BMD hip by DXA Incident fractures by x-ray and hospital records	Change BMD in PAD vs no PAD: -0.60% vs -0.32% (*P *< 0.001 PAD and non-vert fracture risk: HR = 1.47 (95% CI 1.07 to 2.04)	3
Hak, 2000 [3]	Population-based (9)	236 (100%)	No (100%)	No	49	AC by X-spine	MCA by radiogrammetry	MCA in patients with AC progression vs no AC progression -3.5 mm vs -2.0 mm (*P *< 0.01)	3
Samelson, 2007 [12]	Population-based (21)	2,499 (58%)		No	61	AC by X-spine	Incident hip fracture by hospital records and death certificates	Women: HR: 1.4 (0.8 to 2.3) Men: HR: 1.2 (0.2 to 5.7)	4
Bagger, 2006 [1]	Population-based (7.5)	2,262 (100%)	Yes (100%)	No	65	AC by X-spine	BMD lumbar spine and hip and incident fractures by hospital records or X-ray	Change hip BMD AC score ≥3 vs <3: -0.38% vs -0.25% (*P *< 0.001) AC and hip fracture: OR: 2.3 (95% CI 1.1 to 4.8) AC and vert fracture: OR: 1.2 (95% CI 1.0 to 1.5)	4
Schulz, 2004 [17]	Clinic-based (8)	228 (100%)	Yes	No	65.2	AC by CT-scan of spine	BMD spine by CT-scan	Change BMD AC vs no AC: -5.3% vs -1.3% (*P *< 0.001)	6

#adjusted for confounders; NA, not available; AC, aortic calcification; BMD, bone mineral density; DXA, dual-energy x-ray absorptiometry; PAD, peripheral arterial disease; ABI, ankle brachial index; MCA, metacarpal cortical area.

**Table 2:** Prospective studies investigating relationship low BMD and CV disease

Study	Study population (years follow-up)	Number of cases (% women)	Postmenopausal women	CV disease excluded	Mean age (years)	Race	Outcome osteoporosis	Outcome CV disease	Results #	Quality (x nee)
Mussolino, 2007 [69]	Population-based (9)	5,272 (NA)	NA	Yes	60.9 to 69.4	Caucasian (NA%), black and Mexican-American	BMD proximal femur by DXA	CV and stroke mortality by death certificates	Women: BMD and CV mortality RR: 1.26 (95% CI 0.88 to 1.80) BMD and stroke mortality: RR: 1.34 (95% CI 0.86 to 2.07) Men: BMD and CV mortality: RR: 1.05 (95% CI 0.79 to 1.39) BMD and stroke mortality: RR; 0.73 (95% CI 0.43 to 1.23)	3
Farhat, 2007 [6]	Population-based (5.4)	2,310 (55%)	Yes	Yes	73.5	Caucasian (58%) and black	BMD total hip, femoral neck and trochanter by DXA BMD spine by CT-scans	Incident CV disease by hospital records and death certificates	Women: BMD fem neck and incident CV disease: HR: 1.24 (95% CI 1.02 to 1.52) Men: BMD fem neck and incident CV disease: HR: 1.04 (95% CI 0.89 to 1.21)	3
Tamaki, 2009 [75]	Population-based (10)	609 (100%)	Yes (60%)	No	55.9	Japanese	BMD lumbar spine and total hip by DXA	IMT values	<10 YSM: IMT OP vs normal bone mass: 1.55 vs 1.19 (*P *< 0.05) ≥YSM: IMT OP vs normal bone mass: 1.53 vs 1.28 (*P *< 0.05)	3
Browner, 1991 [5]	Population-based (2.8)	9,704 (100%)	Yes	No	NA	Caucasian (99%) and Asian	BMD distal radius, prox radius and calcaneus by single photon absorptiometry	Overall mortality and CV mortality by death certificates	BMD and risk overall mortality: RR: 1.22 (95% CI 1.01 to 1.47) BMD and stroke mortality: RR: 1.75 (95% CI 1.15 to 2.65) BMD and CV mortality: RR: 1.17 (95% CI 0.92 to 1.51)	3
Trone, 2007 [68]	Population-based (7.6)	1,580 (60%)	Yes (NA %)	No	71.9	Caucasian	Prevalence vertebral fracture by lateral spine radiographs	Overall mortality by death certificates	Women: prevalent vertebral fracture and overall mortality: HR: 1.15 (95% CI 0.83 to 1.59) Men: prevalent vertebral fracture and overall mortality: HR: 0.98 (95% CI 0.55 to 1.46)	3
Kado, 2000 [64]	Population-based (3.5)	6,018 (100%)	Yes	No	76.5	Caucasian	BMD total hip by DXA	Overall and CV mortality by death certificates	BMD and overall mortality: RH: 1.3 (95% CI 1.1 to 1.4) BMD and CV mortality: RH: 1.3 (95% CI 1.0 to 1.9)	4
Trivedi, 2001 [67]	Population-based (6.7)	1,002 (0%)	No women included	No	69.7	NA	BMD total hip by DXA	Overall and CV mortality by death certificates	BMD and overall mortality: RR: 0.79 (95% CI 0.65 to 0.97) BMD and CV mortality: RR: 0.72 (95% CI 0.56 to 0.93)	4
Tanko, 2005 [76]	Clinic-based (4)	2,576 (100%)	Yes	No	66.5	NA	BMD lumbar spine and femoral neck by DXA	Incidence CV events self-reported and confirmed by primary documents	HR: 3.9 (95% CI 2.0 to 7.7)	4
Pinheiro, 2006 [66]	Population-based (5)	208 (100%)	Yes	No	75.1	Caucasian	BMD lumbar spine, femoral neck and trochanter by DXA	Overall and CV mortality by death certificates	BMD and overall mortality: HR: 1.44 (95% CI 1.06 to 2.21) BMD and CV mortality: HR: 1.28 (95% CI 1.08 to 2.26)	4
Johansson, 1998 [7]	Population-based (7)	1,468 (56%)	Yes	No	74.0	Caucasian	BMD calcaneus by DPA	Overall mortality by death certificates	Women: RR: 1.19 (95% CI 1.02 to 1.39) Men: RR: 1.23 (95% CI 1.10 to 1.41)	4
Mussolino, 2003 [65]	Population-based (18.5)	3,402 (NA)	NA	Yes	NA	Caucasian (87%) and black	BMD phalangeal by single photon absorption	Stroke mortality by death certificates	Women: RR: 1.01 (95% CI 0.86 to 1.19) Men: RR: 1.13 (95% CI 0.93 to 1.38) Blacks: RR : 0.93 (95% CI 0.72 to 1.21)	4
Samelson, 2004 [70]	Population-based (30)	2,059 (60%)	Yes (85,3-94%)	Yes	60.2	NA	Second MCA by radiogrammatry	Incidence coronary heart disease by hospital records and death certificates	Women: HR: 0.73 (95% CI 0.53 to 1.00) Men: HR: 1.14 (95% CI 0.84 to 1.56)	4
Kiel, 2001 [77]	Population-based (25)	554 (66%)	NA	No	54.4	NA	Second MCA by radiogrammetry	AC by radiograph of the lumbar spine	Women: Sign association % change in MCA and change AC index (*P *= 0.01) Men: No association % change MCA and change AC index (*P *= 0.50)	4
Browner, 1993 [62]	Population-based (1.98)	4,024 (100%)	Yes	Yes	NA	Caucasian	BMD distal radius and calcaneus by single photon absorptiometry	Incident strokes by hospital records and death certificates	HR: 1.31 (95% CI 1.03 to 1.67)	5
Von der Recke, 1999 [8]	Clinic-based (17)	1,063 (100%)	Yes	Yes	50 and 70	NA	BMD distal forearm by single photon absorptiometry with ^125^I source	CV mortality by death certificates, hospital records and autopsy reports	Early menopause: RR: 2.3 (95% CI 1.0 to 5.3) Late menopause: RR: 1.3 (95% CI 0.9 to 1.8)	5
Silverman, 2004 [71]	Clinic-based (3)	2,565 (100%)	Yes	No	67	Caucasian (95.8%)	Prevalence vertebral fracture by lateral spine radiographs	Incident CV event self-reported and confirmed by primary documents	CV event rate women with prevalent vertebral fracture vs no vertebral fracture: 15.1 vs 8.3 (*P *= 0.55)	5
Varosy, 2003 [73]	Clinic-based (4.1)	2,763 (100%)	Yes	Yes	NA	NA	Prevalent and incident skeletal fracture self-reported. Incident fractures were confirmed by radiological reports	Incident coronay event by hospital records	HR: 0.75 (95% CI 0.57 to 0.98)	5
Gonzales-Macias, 2009 [63]	Clinic-based (3)	5,201 (100%)	Yes	No	72.3	Caucasian	eBMD calcaneus by QUS	Overall and CV mortality by medical records	eBMD and overall mortality: HR: 1.19 (95% CI 0.97 to 1.45) eBMD and CV mortality: HR: 1.39 (95% CI 1.15 to 1.66)	6

#adjusted for age; AC, aortic calcification; BMD, bone mineral density; DPA, dual photon absorptiometry; DXA, dual-energy x-ray absorptiometry; IMT, intima media thickness; MCA, metacarpal relative cortical area; NA, not available; QUS, quantitative ultrasonography; YSM, years since menopause.

### Statistical analysis

A formal meta-analysis of the prospective studies investigating the association between bone mass and risk for cardiovascular events and mortality was not possible due to extended heterogeneity between studies with respect to the study population and methods used. Furthermore, the number of prospective studies that were eligible for pooling was too small for analysis. For this reason, narrative summaries are provided in the results section and quantitatively presented in Tables 1 and 2. The heterogeneity between studies in terms of study population and outcome measures is shown in Tables 1 and 2. Moreover, cross-sectional studies are shown in Table 3.

**Table 3:** Cross-sectional studies investigating relationship CV disease and low BMD

Study	Study population	Number of cases	% women	Outcome bone mass	Outcome CV disease	Main results #
Frye, 1992 [35]	Population-based	200	100%	BMD lumbar spine and hip by single photon absorptiometry	AC by x-ray	Association AC and BMD lumbar spine: β-2.213 (*P *< 0.05) Association AC and BMD hip: β-0.661 (NS)
Barengolts, 1998 [32]	Clinic-based	45	100%	BMD lumbar spine and hip by DXA	Coronary calcium score by EBT	Correlation BDM hip and calcium score: r-0.34 (*P *= 0.022) Correlation BMD spine and calcium score: r-0.28 (*P *= 0.056)
Jorgensen, 2001 [27]	Clinic-based	63	52%	BMD femoral neck by DXA	Incident stroke	Women: OR: 6.6 (95% CI 1.8 to 24.8) Men: OR: 0.6 (95% CI 0.1 to 2.3)
Aoyagi, 2001 [40]	Population-based	524	100%	BMD distal and proximal radius, calcaneus single photon absorptiometry by sinlge photon absorptiometry	AC by x-ray	BMD distal radius and AC: OR: 1.1 (95% CI 0.9 ro 1.3) BMD calcaneus and AC: OR: 1.1 (0.9 to 1.3)
Van der Klift, 2002 [29]	Population-based	5,268	57%	BMD lumbar spine and hip by DXA	PAD by ABI	Women: PAD and BMD hip: OR: 1.35 (95% CI 1.02 to 1.79) Men: PAD and BMD hip: OR: 0.89 (95% CI 0.64 to 1.23)
Tanko, 2003 [39]	Population-based	963	100%	BMD hip and lumbar spine by DXA	AC by x-ray	AC and BMD hip: β-0.10, 9 (*P *= 0.004)
Hirose, 2003 [56]	Clinic-based	7,865	9%	OSI calcaneus	baPWV	Women: β-0.11 (*P *< 0.01) Men: β-0.07 (*P *< 0.01)
Pennisi, 2004 [50]	Clinic-based	36	44%	BMD total body, lumbar spine, and hip by DXA and calcaneus by QUS	IMT and presence of plaque in carotid artery	63% patients with BMD spine T <-1 93% patients with BMD hip T <-1
Jorgensen, 2004 [47]	Population-based	5,296	52%	BMD distal radius by single x-ray absorptiometry	IMT and prevalent plaque	BMD and IMT: NS BMD and prevalent plaque: OR: 0.90 (95% CI 0.75 to 1.07) BMD and echogenic plaque: OR: 0.51 (95% CI 0.31 to 0.83)
Montalcini, 2004 [49]	Clinic-based	157	100%	BMD calcaneus by QUS	IMT	BMD and IMT: NS
Magnus, 2005 [23]	Population-based	5,050	36%	BMD hip by DXA	Self reported CV events	Women: OR: 1.22 (0.80 to 1.86) Men: OR: 1.39 (95% CI 1.03 to 1.87)
Bakhireva, 2005 [31]	Population-based	366	51%	BMD lumbar spine and hip by DXA	CAC by CT scan	Women: BMD hip and CAC: OR: 0.69 (95% CI 0.51 to 0.93) Men: BMD hip and CAC: OR: 1.03 (0.75 to 1.41)
Wong, 2005 [30]	Population-based	3,998	50%	BMD lumbar spine and hip by DXA	PAD by ABI	Per SD increase in ABI sign associated with hip BMD: 0.5 (95% CI 0.02 to 0.9)
Yamada, 2005 [53]	Clinic-based	260	59%	BMD lumbar spine by DXA and OSI calcanues	IMT carotid artery and femoral artery	BMD lumbar spine and FA-IMT: ρ-0.117 (*P *< 0.005)
Farhat, 2006 [34]	Population-based	490	100%	vBMD spine by CT scan	AC and CAC by CT scan	AC and BMD: OR: 1.68 (95% CI 1.06 to 2.68) CAC and BMD: OR: 1.19 (95% CI 0.81 to 1.74)
Farhat, 2006 [19]	Population-based	1,489	51%	BMD hip by DXA vBMD lumbar spine by QCT	Prevalent CV disease self reported Prevalent PAD by ABI	Women: Prevalent CV disease and BMD hip: OR: 1.22 (95% CI 1.03 to 1.43) PAD and BMD hip: NS Men: Prevalent CV disease and BMD hip: NS PAD and BMD hip: OR: 1.39 (95% CI 1.03 to 1.84)
Yamada, 2006 [54]	Population-based	149	100%	BMD lumbar spine by DXA and vBMD calcaneus by QCT	IMT and PWV	FA-IMT and BMD spine: β-0.067 (*P *< 0.05) PWV and BMD spine: NS
Sumino, 2006 [60]	Clinic-based	315	100%	BMD lumbar spine by DXA	baPWV	Association baPWV and BMD: β-0.265 (*P *= 0.002)
Sinnot, 2006 [43]	Clinic-based	480	65%	BMD lumbar spine by QCT	Calcium score by CT-scan	No correlation CAD and BMD in women and men
Shaffer, 2007 [51]	Population-based	870	61%	BMD lumbar spine, hip and distal radius by DXA	IMT	Women >60 years: IMT and BMD spine: β-73.0 (*P *< 0.001) IMT and BMD hip: β-62.4 (*P *< 0.001) Men >60 years: IMT and BMD radius: β-27.0 (*P *< 0.001)
Sumino, 2007 [61]	Clinic-based	85	100%	BMD lumbar spine by DXA	Brachial arterial endothelial function (FMD)	Correlation FMD and BMD: r .034 (*P *< 0.01) Association FMD and BMD: β 0.40 (*P *< 0.01)
Hyder, 2007 [36]	Clinic-based	365	64%	BMD lumbar spine by CT-scan	Atherosclerotic calcium in carotid, coronary and iliac arteries by CT-scan	Women: Calcium score aorta and BMD: OR: 3.14 (95% CI 1.55 to 6.38) Calcium score iliac arteries and BMD: OR: 2.20 (95% CI 1.13 to 4.29) Men: Calcium score carotid and BMD: OR: 2.85 (95% CI 1.02 to 7.96) Calcium score aorta and BMD: OR: 5.90 (95% CI 1.78 to 19.6)
Shen, 2007 [42]	Population-based	682	56%	BMD lumbar spine and hip by DXA	CAC by CT scan	CAC and BMD spine: -0.105 ± 0.132 (NS) CAC and BMD hip: 0.022 ± 0.142 (NS)
Sioka, 2007 [24]	Clinic-based	21	0%	BMD lumbar spine and hip by DXA	CAD by angiography	BMD in severe CAD vs no CAD: 77.8% vs 37.5%, *P *=?
Sumino, 2008 [52]	Clinic-based	175	100%	BMD lumbar spine by DXA	IMT	BMD and IMT β-0.313 (*P *= 0.001)
Kim, 2008 [48]	Clinic-based	194	100%	BMD lumbar spine and hip by DXA Prevalent vertebral fracture	IMT and prevalent plaque	BMD and IMT: NS BMD and plaque: NS Vertebral fracture and plaque: OR: 2.8 (95% CI 1.17 to 7.12)
Frost, 2008 [45]	Clinic-based	54	100%	Lumbar spine and hip by DXA	IMT and PWV	BMD spine and IMT: r -.025 (*P *= 0.26) BMD hip and IMT: r-0.17 (NS) BMD and PWV: NS
Mangiafico, 2008 [57]	Clinic-based	182	100%	BMD lumbar spine and hip DXA	PWA (AIx and PWV)	BMD hip and AIx: β-5.46 (*P *< 0.0001) BMD spine and Aix: β-3.29 (*P *< 0.0001)
Tekin, 2008 [25]	Clinic-based	227	100%	BMD lumbar spine by DXA	Prevalence CAD	CAD and low BMD: OR: 0.68 (95% CI 0.39 to 1.28)
Broussard, 2008 [18]	Population-based	3,881	51%	BMD total femur by DXA	Framingham CHD risk score by Framingham CHD prediction model	Women: moderate CHD risk and low BMD: OR: 1.45 (95% CI 1.03 to 2.06) high CHD risk and low BMD: OR: 1.73 (95% CI 1.12 to 2.66) Men: NS
Chow, 2008 [41]	Population-based	693	54%	vBMD lumbar spine and hip by QCT and vBMD distal radius by HRpQCT	AC by CT-scan	Women: NS Men: NS
Hyder, 2009 [37]	NA	1,909	50%	vBMD lumbar spine by CT scan	CAC and AAC score	Women: vBMD and CAC (*P*-trend <0.002) vBMD AND AAC (*P*-trend <0.004) Men: vBMD and CAC (*P*-trend <0.034) vBMD and AAC (*P*-trend <0.001)
Hmamouchi, 2009 [46]	Clinic-based	72	100%	BMD lulmbar spine and hip by DXA	IMT in carotid artery and femoral artery	CA-IMT and BMD hip: r-0.330 (*P *< 0.05) FA-IMT and BMD hip: NS IMT and BMD lumbar spine: NS
Mikumo, 2009 [58]	Clinic-based	143	100%	BMD lumbar spine by DXA	PWV	BMD and PWV: r-99.78 (NS)
Marcowitz, 2005 [20]	Clinic-based	209	88%	Lumbar spine, hip and distal radius by DXA	CAD	Osteoporosis: OR: 5.58 (95% CI 2.59 to 12.0) for CAD
Ness, 2006 [38]	Clinic-based	1,000	100%	Diagnosis osteoporosis or osteopenia by electronic medical records	AVD	Prevalence AVD osteoporotis vs osteopenia: 60% vs 35% (*P *< 0.001) Prevalence AVD osteoporis vs normal bone mass: 60% vs 22% (*P *< 0.001)
Gupta, 2006 [78]	Clinic-based	101	100%	BMD lumbar spine and total hip by DXA	Prevalent CV disease	Prevalent CV disease in low BMD vs normal BMD: 61% vs 38% (*P *< 0.025)
Mangifico, 2006 [28]	Clinic-based	345	100%	BMD lumbar spine and femoral neck by DXA	PAD by ABI	PAD and BMD lumbar spine: OR: 1.01 (95% CI 0.97 to 1.05) PAD and BMD hip: OR: 0.20 (95% CI 0.05 to 0.70)
Erbilen, 2007 [33]	Clinic-based	74	0%	BMD lumbar spine and hip by DXA	CAD	Association BMD and CAD: OR: 5.4 (95% CI 1.66 to 17.49)
Sennerby, 2007 [21]	Clinic-based	1,327	100%	Incident hip fracture by X-ray and hospital record	Prevalent CV disease by questionnaire	OR: 2.38 (95% CI 1.92 to 2.94)
Varma, 2008 [22]	Clinic-based	198	74%	Lumbar spine and hip by DXA	Obstructive CAD	Prevalence CAD osteoporosis vs osteopenia: 76% vs 68% (*P *< 0.01) Prevalence CAD osteoporosis vs normal bone mass: 76% vs 47% (*P *< 0.005)
Seo, 2009 [59]	Clinic-based	253	100%	BMD lumbar spine and hip by DXA	baPWV	Sign association BMD hip and baPWV: Β-0.123 (*P *< 0.05)
Pouwels, 2009 [16]	Clinic-based	6,763	73%	Incident hip fracture	Incident stroke by ICD 9 code	Risk hip fracture after stroke Women: OR: 2.12 (95% CI 1.73 to 2.59) Men: OR: 1.63 (95% CI 1.17 to 2.28)

#adjusted for confounders; BMD, bone mineral density; AC, aortic calcification; DXA, dual-energy x-ray absorptiometry; PAD, peripheral arterial disease; ABI, ankle brachial index; OSI, osteosono assessment index; baPWV, brachial-ankle pulse wave velocity; IMT, intimal medial thickness; CAC, coronary artery calcium; QCT, quantitative computerized tomography; PWV, pulse wave velocity; CAD, coronary artery disease; PWA, pulse wave analysis; AIx, augmentation index; CHD, coronary hearth disease; AVD, atherosclerotic vascular disease.

## Results

### Studies included

**Figure 1:**
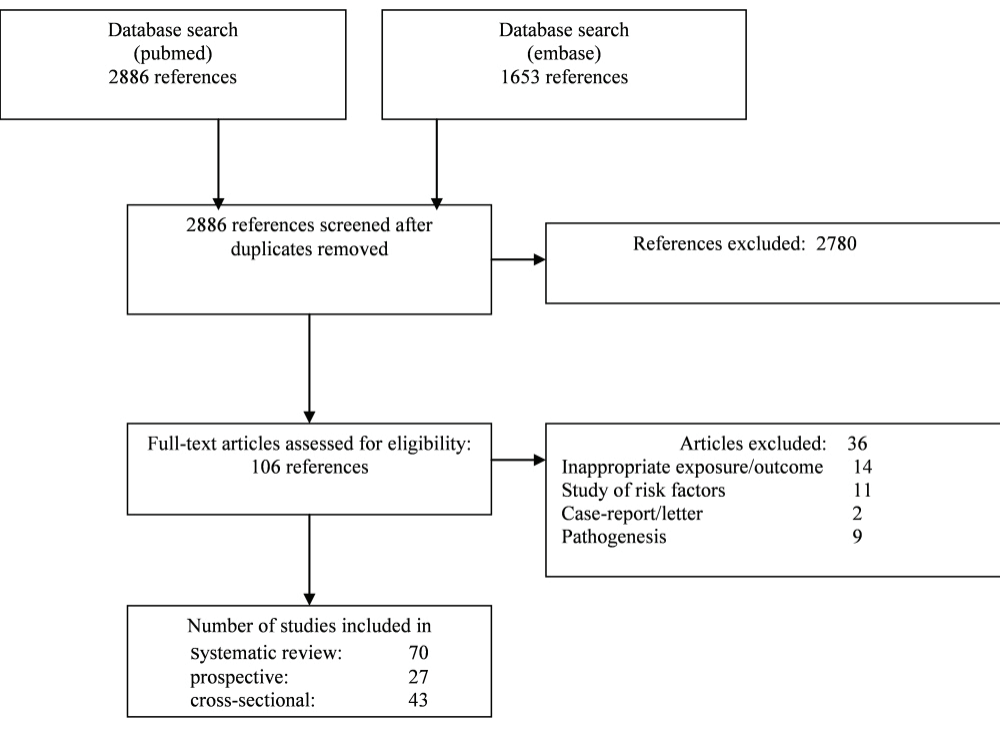
**Flow-chart of the systematic review**.

Our search strategy resulted in 2,886 references. The search strategy resulted in 70 relevant articles, including 9 studies prospectively assessing the relationship between CV disease and osteoporosis and 18 prospective studies about the inverse relationship. Figure 1 shows the flow-chart of included and excluded studies.

### Study results

#### The relationship between CV disease and osteoporosis

##### Cardiovascular disease and fracture risk

Seven population-based cohort studies assessed the relationship between CV disease and fracture risk [1,2,4,12-15] (Table 1). An increased risk of incident fractures was observed in four studies with risk rates ranging from 1.2 to 6.7 [1,2,13,14].

The largest study included more than 30,000 twins with a follow-up duration of 20 years [13]. In this study, twins, without prevalent CV disease, were included at the age of 50 years and followed up until a first hip fracture, death or end of follow-up period. Twins were considered unexposed until the first CV event. An increased hip fracture risk was found after all diagnoses of CV disease in both men (hazard ratio (HR) 6.65; 95% CI 4.82 to 9.19) and women (HR 4.42; 95% CI 3.49 to 5.61).

Furthermore, this study showed that CHD was associated with an increased fracture risk (HR 2.32; 95% CI 1.91 to 2.84) as was cerebral vascular disease (HR 5.09 95% CI 4.18 to 6.20) [13]. This was confirmed in a large population case-control study. This case-control study was conducted using the Dutch PHARMO Record Linkage System database. Patients (*n *= 6,763) with a hip fracture were compared with age- and sex-matched patients without a hip fracture (*n *= 26,341), with the objective to evaluate the association between stroke and risk of hip fracture [16]. The prevalence of stroke was 3.3% in cases versus 1.5% in control patients. The risk for a hip fracture was increased in patients who experienced a stroke before the index date (OR 1.96; 95% CI 1.65 to 2.33).

Three studies looked at the association between PAD and fracture risk. PAD was associated with increased risk for non-vertebral fractures (HR 1.47; 95% CI 1.07 to 2.04) [2] and hip fractures (HR 3.20; 95% CI 2.28 to 4.50) [13]. In contrast, a smaller study in men and women, with shorter follow-up time, did not find an association between PAD and non-vertebral fracture risk [15]. Time of follow-up might be an important factor explaining different results, for the risk of fractures was highest more than 10 years after the diagnosis of PAD [13].

Longitudinal analysis in healthy postmenopausal women (*n *= 2,262) showed that aortic calcifications (AC) represented a strong predictor for fragility fractures: AC predicted a 2.3-fold increased risk for hip fracture [1]. Not only women, but also men with advanced AC have a two- to three-fold increased fracture risk [14]. However, a large population-based study with 21 years follow-up, found no evidence that severity of vascular calcification, measured as AC, is associated with an increased risk of incident hip fracture [12]. Conflicting results might be due to differences in population and methodology. The incident fracture rates were equal in comparison to the other studies.

Hence, although heterogeneity makes it difficult to draw firm conclusions, there is evidence that subjects with atherosclerotic disease are at an increased risk for frailty fractures. There are insufficient data to draw conclusions about fracture risk in patients with prevalent coronary or cerebral CV disease.

##### Cardiovascular disease and bone loss

Longitudinal data about CV disease and bone loss were available from six studies [1-4,15,17]. All studies showed that prevalent CV disease was associated with an increased bone loss during follow-up, independent of age and traditional risk factors. In addition, several cross-sectional studies similarly reported that prevalent CV disease is associated with low BMD [18-22]. In the next section the results are presented per subcategory of CV disease.

The association of CHD and BMD was only addressed in cross-sectional studies and all but one found an association with low BMD [20,22-25]. Several studies reported increased bone loss after an incident stroke. Particularly patients who are wheelchair-bound or have paretic limbs as a result of the stroke have significant bone loss within months after the stroke [26]. These studies were not included in this review, for the underlying pathogenesis is obvious. One study looked at bone density immediately after the stroke and found that female stroke patients have lower BMD than controls [27]. Since the BMD measurement was assessed within six days after the stroke, one may assume that the possible differences are not a result of immobilisation.

A large prospective study found that men with prevalent PAD had an increased rate of hip bone loss compared with men without PAD (-0.6% vs -0.3%, *P *< 0.001) [2]. In another, smaller, study the association between PAD and bone loss in women was weaker and not observed in men [15]. In addition, a number of cross-sectional studies showed that women and/or men with PAD have decreased BMD [19,28-30].

Numerous reports have looked at the association between subclinical atherosclerosis and osteoporosis. Men and women with progression of AC have significantly higher bone loss in the lumbar spine compared with subjects without AC progression (-1.5% vs 1.4%) [4]. This is in line with other studies where AC progression is associated with higher rates of bone loss in the proximal femur and metacarpal bones [1,3]. Furthermore, several studies confirmed the prospective data and showed that subjects with calcifications in the aorta, coronary arteries, carotid arteries or femoral arteries have significant lower BMD compared with controls [31-39]. Only a few studies fail to find an association [40-43]. In recent years, many studies have examined the association between atherosclerosis and osteoporosis. An increased IMT has been associated with severity of atherosclerosis and increased cardiovascular risk and considered useful in identifying subjects with increased risk [44]. An association between IMT and BMD was studied intensively and most of the studies reported an association of increased IMT with low bone density [45-54]. Endothelial dysfunction is considered to be an early phase of atherosclerosis and one way to measure this is to focus on arterial compliance. The endothelium plays an important role in determining vascular tone and dysfunction will result in increased arterial stiffness [55]. In line with earlier discussed results, an increased arterial stiffness is associated with low BMD [45,54,56-61].

Altogether, the results strongly suggest that subjects with subclinical atherosclerosis and early CV disease are at increased risk of bone loss. Again, there were insufficient data to reach conclusions about bone loss in patients with prevalent coronary or cerebral CV disease.

#### The relationship between osteoporosis and CV disease

Eighteen studies, most of moderate quality, reporting about the relationship between osteoporosis and CV disease were included. Results will be discussed per subcategory of CV disease, when possible.

##### Low bone mineral density and cardiovascular mortality

The association of osteoporosis with CV mortality was studied in 10 prospective studies [5,7,8,62-68] (Table 2). Low bone mass was inversely related with CV mortality in seven studies [5,7,8,62-64,66,67]. Postmenopausal women with a low BMD had a 1.2- to 2.3-fold increased risk of dying from CV events, independent of traditional CV risk factors [7,8,66]. Similar results were found in elderly men [7,67]. Studies in postmenopausal women with relative short follow-up periods (around three years) showed no or minimally significant elevated mortality rates [5,63,64]. Two large population-based studies in elderly men and women did not reveal a significant association between low bone mass and CV mortality [65,69]. The most recent and largest study determined the risk of CV mortality in 5,272 persons [69]. Women with low BMD had higher risk for CV mortality; however, this did not reach significance (relative risk (RR) 1.26; 95% CI 0.88 to 1.80). No association was found in men.

Focusing on the few studies that reported the results per CV subcategory, women with low bone mass had no or a small increased risk for mortality by coronary heart disease (RR 1.17; 95% CI 0.92 to 1.51) and (relative hazard 1.3; 95% CI 1.0 to 1.8), respectively [5,64] and two out of three studies showed that men and women with low BMD had a 1.3- to 1.7-fold increased risk for stroke mortality [5,62,65].

##### Low bone mineral density and incident cardiovascular disease

A total of six studies assessed the risk of incident CV events in persons with osteoporosis [6,62,70-73]. Most of them show a significant inverse relationship between BMD and incident CV events in women (HR 1.23 to 3.9) [6,39,62,70] but not in men [6,70]. Two studies related the prevalence of vertebral fractures with future CV events and were unable to find any association [68,71]. Surprisingly, one study showed that women with prevalent fractures and known CHD had a reduced risk for CV events [73].

Few articles assessed incident CV events separated per CV category. Three studies assessed the risk for CHD. Two studies showed an association with increased risk for CHD in postmenopausal women [72,73]. One study could not find an association in elderly men and women [70]. Cerebrovascular events were studied in two articles. Both found an increased risk for stroke in postmenopausal women with low BMD with hazard ratios of 1.31 and 4.1 [62,72].

There was a considerable heterogeneity in measurement of osteoporosis. It is shown that the specificity and sensitivity of the densitometry tests differs greatly, and the site of measurement plays an important role in diagnosing osteoporosis as well [74]. Only six studies used dual energy absorptiometry (DXA) measurements to assess BMD [6,64,66,67,69,75,76], while in the other studies BMD was measured with older techniques such as single photon absorptiometry, dual photon absorptiometry (DPA) or quantitative ultrasonography (QUS). Most studies measured BMD of the hip and lumbar spine, but also distal radius and heel were measured and in some the phalangeals.

##### Low bone mineral density and subclinical atherosclerosis

In addition to associations with CV events, low BMD has also been shown to be associated with surrogate markers of CV disease, such as vascular calcification. In women with the largest decrease in metacarpal cortical area during a 25-year follow-up, the most severe progression of aortic calcification was observed [77] and women with a prevalent vertebral fracture had a higher IMT measured 10 years later [75]. Moreover, results from several cross-sectional studies confirmed that both women and men with low bone mass, compared to subjects with normal bone mass, have significantly more subclinical atherosclerosis [20,28,31-34,37,38,45,48,49,51,52,78,79], increased risk of peripheral arterial disease [28,29,34,54] and other surrogate end markers for CV disease [57,60,61].

Taken together, there is some evidence that persons with low BMD are at increased risk for CV events and subsequent CV mortality. However, variations in study design, for example, study population and outcome measures, limits interpretation. Since only a few studies assessed the CV outcome divided per CV subcategory, no conclusions can be drawn concerning a relationship between osteoporosis and specific categories of CV disease.

### Links between CV disease and osteoporosis

#### Common pathogenesis

**Figure 2:**
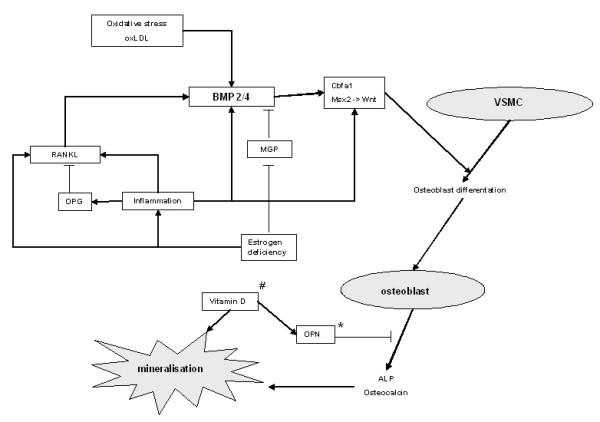
**Vascular calcification**. Vascular calcification is an active process regulated by factors known to be involved in the process of osteogenesis. Vascular smooth muscle cells are able to differentiate towards osteoblast-like cells, promoted by a variety of stimuli, including BMP, RANKL, oxidative stress, inflammation and estrogen deficiency. These osteoblastic cells produce osteocalcin and ALP, important factors in mineralisation. ^# ^Excessive vitamin D promotes mineralisation. * It is not clear whether OPN promotes or inhibits calcification in the arterial wall, in bone mineralisation it is a known mineralisation inhibitor. Abbreviations: ALP, alkaline phosphatase; BMP, bone morphogenetic protein; Cbfa1, core binding factor-α1; MGP, matrix GLA protein; Msx2, msh homeobox 2; OPG, osteoprotegerin; OPN, osteopontin; ox-LDL, oxidized low density lipoprotein; RANKL, receptor activator of nuclear factor-B ligand; VSMC, vascular smooth muscle cell; Wnt, combination of wingless and Int.

CV disease is preceded by atherosclerosis, for example, arterial disease. Atherosclerosis is a long-term process in which deposits of cholesterol, cellular waste products and calcium accumulates in the arterial wall causing it to thicken. Clinically, atherosclerosis is manifested by coronary heart disease, cerebrovascular disease and peripheral arterial disease. Endothelial dysfunction is the first step in the pathogenesis of atherosclerosis and predicts future CV events [80]. Calcification in the aorta and coronary arteries, for example, vascular calcification, may be a surrogate marker for atherosclerosis and increased CV risk [81]. In a recent meta-analysis patients with calcifications were found to have an increased risk for CV mortality and events [10]. Presently, vascular calcification is regarded as an active process, regulated by factors known to be involved in the process of osteogenesis, such as bone morphogenetic protein (BMP), alkaline phosphatase (ALP), osteopontin (OPN) and matrix GLA protein (MGP) [82-85] (Figure 2). Accumulating evidence suggests that calcification is a consequence of active bone formation by osteoblast-like cells [86]. Vascular smooth muscle cells (VSMCs) are able to re-differentiate towards osteoblast-like cells and a subpopulation, that is, calcifying vascular cells (CVCs), were shown to form nodules and mineralisation spontaneously [87]. *In vitro*, these osteoblastic cells produce hydroxyapatite, a mineral important in bone formation [88]. In the following paragraphs some of the bone-related factors that are involved in vascular calcification will be discussed in more detail.

BMPs are members of the transforming growth factor-β superfamily and important factors in the regulation of osteoblast differentiation. BMP acts through upregulation of transcription factors important in bone metabolism, such as core binding factor-α1 (Cbfα1), also known as runt-related transcription factor 2 (Runx2), and msh homeobox 2 (Msx2). BMP appears to be an important mediator in vascular calcification. An increased expression of BMP2 and BMP4 is found in atherosclerotic lesions in endothelial cells, foam cells and VSMCs [88,89]. *In vitro *studies showed that several factors that are known to induce CV disease, such as oxidative stress, oxidized low-density lipoprotein (ox-LDL) and tumor necrosis factor alpha (TNF-α), are able to upregulate BMP expression in endothelial cells [90,91].

MGP is a calcium-binding protein and requires vitamin K to function. MGP is found to be expressed in areas with arterial calcification [92] and may be an important calcification inhibitor. MGP knock-out mice developed extensive calcification in coronary arteries [93]. Recently the mechanism by which MGP inhibits calcification has become clear. *In vitro*, MGP has been shown to inhibit calcification by binding to BMP2, thereby blocking the induction of osteoblasts [94].

OPN is a glycoprotein that accumulates in the extracellular matrix of bone tissue where it binds to hydroxyapatite and calcium. In bone, OPN is expressed by (pre-) osteoblasts and osteoclasts and is also found to be highly expressed in the atherosclerotic artery [89,92]. Whether it promotes or inhibits calcification in the arterial wall is not completely clear [95]. While high OPN serum levels are associated with vascular calcification [96] and vitamin increases OPN and subsequent calcification in bovine VSMC's [97], OPN is also shown to inhibit calcification by inhibiting *de novo *hydroxyapatite production [98].

ALP is found on the surface of osteoblasts and is often used as a marker for bone turnover. ALP is an enzyme that catalyses the hydrolysis of phosphate esters. Hydrolysis of pyrophosphate, which is an inhibitor of hydroxyapatite formation, is especially needed to facilitate normal mineralisation [99]. *In vitro *studies in VSMC's showed that the ALP expression is increased in response to inflammatory markers, LDL and oxidative stress and this increased expression was associated with increased mineralisation [100-102].

The recent identification of receptor activator of nuclear factor-kB (RANK), osteoprotegerin (OPG) and RANK ligand (RANKL) provides more insight into bone metabolism [103]. Most interestingly, there is increasing evidence that OPG is a key regulator in the pathogenesis of osteoporosis and vascular calcification. OPG production by osteoblastic cells is regulated by a number of factors, including BMP-2, inflammation, estrogen, vitamin D and oxidative stress [104]. OPG is expressed in various tissues, including the skeleton and vascular wall, and serves as a soluble decoy for RANKL [105]. Interestingly, OPG knock-out mice show, in addition to early-onset osteoporosis, increased vascular calcification [106]. *In vitro *studies have shown that OPG appears to be important for endothelial cell survival [107] and may inhibit active calcification [108]. Surprisingly, while experimental studies showed that OPG might protect against vascular calcification, OPG levels appear to be elevated in patients with CV disease. Several, but not all, clinical studies found a correlation of high OPG serum levels and more severe CV disease [45,50,62,109-111]. Other pathways interacting with OPG might explain this discrepant finding. Estrogen deficiency results in an increased vascular OPG/RANKL ratio with subsequent increased calcification in an animal model [112]. Furthermore, pro-inflammatory cytokines are shown to elevate OPG levels in patients with CV disease [113]. Thus, while OPG appears to play a role in the pathogenesis of atherosclerosis, the exact mechanism remains to be elucidated.

Another important mechanism linking CV disease and osteoporosis is *Wnt *signalling, a combination of the genes *Wg *(wingless) and *Int*. Animal models showed the important role of *Wnt *signalling in bone formation through lipoprotein receptor-related protein 5 (LRP5), lipoprotein receptor-related protein 6 (LRP6) and β-catenin [114]. *Wnt *signalling is suggested to play an important role in bone formation and bone adaptation to mechanical loading [115,116]. Interestingly, TNF-α [117], oxidative stress [118] and vitamin D [119] are shown to promote vascular calcification through the *Wnt *signalling pathway and this supports the hypothesis that *Wnt *signalling is an interesting new molecular mechanism that influences bone and vascular metabolism.

#### Common risk factors

CV disease and osteoporosis are both common diseases in elderly men and women. While the increased prevalence of both conditions is often attributed to aging, most of the associations found in observational studies remain significant after adjustment for age. Other important traditional risk factors are also shared, such as inactivity, smoking, estrogen deficiency and chronic inflammation, explaining part of the link between CV disease and osteoporosis [9].

Estrogen deficiency is considered an important risk factor for osteoporosis [120] and some studies suggest estrogen deficiency to be a cardiovascular risk factor [121-123]. Estrogen regulates bone turnover and the CV system directly and indirectly through the effects on the immune system, antioxidant system and other risk factors. After menopause, estrogen levels decrease rapidly resulting in an upregulated osteoclast formation and differentiation, inducing high bone turnover and accelerated bone loss [124]. Furthermore, following estrogen withdrawal the production and secretion of the pro-inflammatory cytokines interleukin-6 (IL-6), interleukin-1 and TNF-α is increased [116,125].

Presently, inflammation is considered to play an important role in the process of atherosclerosis [126,127]. Both cellular and humoral pathways of the immune response contribute to an important part in the pathogenesis of atherosclerosis [128]. Markers of inflammation, such as pro-inflammatory cytokines and C-reactive protein (CRP), are involved in the development of atherosclerosis and CRP predicts cardiovascular events independently of other CV risk factors [129,130]. There is accumulating evidence that inflammation influences bone metabolism and is considered to be the most important cause of postmenopausal osteoporosis. Pro-inflammatory cytokines enhance bone resorption directly through an induction of osteoclastogenesis or through the OPG pathway [116,131].

Recent research has identified new common mediators for vascular calcification and bone loss, such as hyperlipidemia, oxidative stress and vitamin D deficiency. An abnormal lipid profile, that is, high levels of total cholesterol, LDL and triglycerides and low levels of high-density lipoprotein (HDL), is known to play a key role in development of atherosclerosis and CV disease [132,133]. Interestingly, HDL is able to regulate the calcification of VSMCs [134]. HDL inhibited the spontaneous and cytokine induced osteogenic differentiation of CVCs *in vitro*. The role of lipids in the regulation of bone mass is more complicated. While experimental studies showed that ox-LDL influences bone metabolism [135], results in observational studies are contradictory [1,136-138].

Oxidative stress is believed to increase with age and is associated with hypertension and atherosclerosis [139]. Free radicals have important effects on osteoclast differentiation and function [140] and oxidative stress markers are significantly associated with BMD [141]. *In vitro*, minimally oxidized low-density lipoprotein (MM-LDL) enhances the differentiation of VSMC's towards osteoblastic cells. Interestingly, antioxidants inhibited these effects [100].

The prevalence of vitamin D deficiency is high among elderly men and women [142] and associated with osteoporosis and increased fracture risk [143]. Observational studies showed an inverse association of vitamin D deficiency with hypertension and CV events, suggesting a role for low vitamin D [144-148]. Proposed mechanisms are effects on myocardial gene expression, the renin-angiotensin axis or through secondary hyperparathyroidism. Important risk factors as physical condition and immobility were rarely assessed. Animal models and *in vitro *studies on the other hand, demonstrated that toxic levels of vitamin D induce vascular calcification [97,149]. Interestingly, osteoprotegerin has been shown to inhibit the vitamin-induced calcifications in an animal model [150]. It has been suggested that vitamin D has a biphasic relation with vascular calcification and that both vitamin D deficiency and vitamin D excess results in increased vascular calcification.

#### Genetic studies

In complex, multifactorial diseases genetic factors are believed to play an important role in the pathogenesis in addition to environmental influences. Identifying candidate genes offers opportunities to gain more insight into possible shared pathogenesis and common risk factors in CV disease and osteoporosis. Many candidate genes have been examined, mainly genes coding for known factors, such as cytokines, bone-associated factors and receptors. The genes that might be involved in both diseases will be discussed here.

Polymorphism in the *IL-6 *gene, a cytokine involved in bone metabolism and CV disease, might be an interestingly candidate gene. A *G174C *polymorphism in the promoter region of the *IL-6 *gene was shown to be associated with low bone mass in the radius in postmenopausal women [151] and with a high blood pressure and increased CV risk in men [152].

*Vitamin D receptor *polymorphisms have been associated in many studies with bone density [153,154]. Although this could not be replicated in a large meta-analysis, it did show that the *Cdx2 *polymorphism was associated with risk for vertebral fractures [155]. In addition, the *BsmI *polymorphism was associated with IMT and myocardial infarction (MI) [156,157], strengthening the possible role of vitamin D in linking CV disease and osteoporosis.

One of the most interesting candidate genes to mention is the *OPG *gene, located on chromosome 8 and several single nucleotide polymorphisms (SNPs) are identified in this gene. So far, studies were able to associate different SNPs with either bone density or vascular disease. SNPs *A163G *and *T245G *were associated with osteoporotic fractures [158]. The linked polymorphisms *T950C *and *C1181C *within the promoter region of the *OPG *gene were associated with an increased risk for CAD in men [159]. In addition, *C1181C *was also associated with first-ever intracerebral haemorrhage [160]. Furthermore, another SNP in the promoter region in the TATA box was related to vascular morphology and function [161].

A genetic defect in the *Wnt *signalling pathway was recently discovered in a family with features of metabolic syndrome and early onset coronary artery disease [162]. This rare mutation in the *LRP6 *gene is associated with dyslipidemia, hypertension and diabetes. This finding supports further research for mutations in genes involved in the *Wnt *signalling pathway.

Collagen type I is an important protein in the mineralisation matrix and connective tissue. Mutations in this gene are associated with low BMD and fracture risk [163]. Interestingly, besides low BMD, individuals with a SNP in the *COL1A *gene (*rs42524*) had an increased prevalence of stroke and MI [164].

The calcium-sensing receptor (CASR) is a receptor involved in the regulation of calcium homeostasis. A SNP in the *CARS *gene (*A986S*) was associated with higher serum calcium and increased prevalence of coronary artery disease (CAD) and MI [165]. This SNP was also associated with low BMD in premenopausal women [166]. However, the role in postmenopausal osteoporosis is not clear, since several studies showed no association of this SNP with BMD or fracture risk in postmenopausal women [167,168].

An interesting candidate gene to mention is the *klotho *gene. Defects in the *klotho *gene have been shown to result in arteriosclerosis and increased IMT in klotho deficient mice [169]. A SNP in this gene (*G395A*) was associated with CAD. Surprisingly, this same SNP was associated with bone density [170] and was suggested to be involved in the pathophysiology of bone loss. This SNP in the promoter region resulted in impaired function of the gene. What makes this gene interesting is that it might offer a new treatment approach, because the abnormalities seen in klotho-deficient mice can be reversed by restoring the klotho expression [171].

Finally, polymorphisms in the apolipoprotein E (*APOE*) gene has been studied intensively. It has been associated with hypertension, atherosclerotic disease and CV disease [172-174]. Furthermore, *APOE *gene polymorphisms have been suggested to be associated with low BMD and fracture risk. However, a recent meta-analysis was unable to show a strong and consistent association with BMD and fracture incidence [175].

## Discussion

Our study is the first to systematically review the epidemiological literature about the association between CV disease and osteoporosis. An extensive literature search yielded 27 prospective studies addressing this relationship. Due to considerable heterogeneity in study design and outcome measurements the results could not be pooled. Focusing on the methodologically strongest studies (those with minimal selection bias and the appropriate assessments, that is, a methodological score of more than 3), our review indicates that the prevalent subclinical CV disease predicts future fractures and bone loss [2-4,13-15] (Table 4).

**Table 4:** Summary of findings in high quality prospective studies

	Association	No association
CV disease and OP	*N *= 6	*N *= 0
Bone mass and CV events	*N *= 3	*N *= 2

Furthermore, there is some evidence that low bone mass predicts CV mortality and CV events [6,62,68,69,75].

Interestingly, several studies demonstrated shared risk factors, supporting the existence of a direct association between vascular calcification and bone biology.

Due to the substantial diversity of patients and study methods, pooled analysis was not considered appropriate. Although numerous efforts were made to investigate the association between CV disease and osteoporosis, a vast majority of studies used secondary outcome measurements, while a limited number of studies used primary outcome measurements such as incident CV events or osteoporosis. Furthermore, the population studied varied with respect to age, sex, baseline risk for CV events or fractures and ethnicity. Larger prospective studies in elderly persons, men and women, are needed to answer this question. To reduce heterogeneity we encourage that in new studies well-defined outcome measures should be incorporated, such as incident CV disease presented per subcategory of CV disease and measurement of BMD by DXA-scans on regular interval periods.

## Conclusions

The current evidence indicates that individuals with prevalent (sub)clinical CV disease are at increased risk for bone loss and subsequent fractures. Presently, no firm conclusions can be drawn to which extent low BMD might be associated with increased cardiovascular risk. Age, estrogen deficiency and inflammation represent the most important common risk factors and the discovery of new pathways, for example, OGP/RANKL and *Wnt *signalling, might provide interesting new therapeutic options. Altogether our results suggest that bone density screening could be recommended in patients with prevalent CV disease.

## Abbreviations

ABI: ankle brachial index; AC: aortic calcifications; ALP: alkaline phosphatase; APOE: apolipoprotein E; BMD: bone mineral density; BMP: bone morphogenetic protein; CAD: coronary artery disease; CASR: calcium-sensing receptor; Cbfa1: core binding factor-α1; CDH: coronary heart disease; CRP: C-reactive protein; CV: cardiovascular; CVC: calcifying vascular cells; DPA: dual photon absorptiometry; DXA: dual energy absorptiometry; HDL: high density lipoprotein; HR: hazard ratio; IL-6: interleukine-6; IMT: intima media thickness; LRP5: lipoprotein receptor-related protein 5; LRP6: lipoprotein receptor-related protein 6; MGP: matrix GLA protein; MI: myocardial infarction; MM-LDL: minimally oxidized low-density lipoprotein; Msx2: msh homeobox 2; OPG: osteoprotegerin; OPN: osteopontin; OR: odds ratio; ox-LDL: oxidized low density lipoprotein; PAD: peripheral arterial disease; QUS: quantitative ultrasonography; RANK: receptor activator of nuclear factor-B; RANKL: receptor activator of nuclear factor-B ligand; RR: relative risk; Runx2: runt-related transcription factor 2; SNP: single nucleotide polymorphism; TNF-α: tumour necrosis factor alpha; VSMC: vascular smooth muscle cell; Wnt: combination of wingless and Int.

## Competing interests

The authors declare that they have no competing interests.

## Authors' contributions

DU conducted the data collection, interpretation and analysis of the data and drafted the manuscript. LT participated in interpretation and analysis of the data and helped to draft the manuscript. WL conceived of the hypothesis of the manuscript and participated in study design and coordination. MT, HR and WL helped to draft the manuscript. All authors critically reviewed, contributed to and approved the final manuscript.

## Supplementary Material

Additional file 1 **Medline search**. Complete medline search on 8 June 2010. Click here for file

Additional file 2 **Quality assessment cohort studies**. List of quality assessment of cohort studies as proposed by the Dutch Cochrane Collaboration. Click here for file
