# Supplementary material for: (Sub)clinical cardiovascular disease is associated with increased bone loss and fracture risk; a systematic review of the association between cardiovascular disease and osteoporosis
Source: Arthritis Res Ther. 2011 Jan 17;13(1):R5. doi: 10.1186/ar3224 (PMC3241350; doi:10.1186/ar3224)
Supplement: Additional file 1 — Medline search. Complete medline search on 8 June 2010. [file ar3224-S1.DOC]

| **Search** | **Most Recent Queries** | **Time** | **Result** |
| --- | --- | --- | --- |
| [#10](http://www.ncbi.nlm.nih.gov/pubmed/?querykey=10&dbase=pubmed&querytype=eSearch&) | Search **#9 NOT #5 NOT #6** | 03:57:57 | [214](http://www.ncbi.nlm.nih.gov/pubmed/?cmd=HistorySearch&querykey=10&) |
| [#9](http://www.ncbi.nlm.nih.gov/pubmed/?querykey=9&dbase=pubmed&querytype=eSearch&) | Search **#1 AND #4 AND #8** | 03:57:36 | [343](http://www.ncbi.nlm.nih.gov/pubmed/?cmd=HistorySearch&querykey=9&) |
| [#8](http://www.ncbi.nlm.nih.gov/pubmed/?querykey=8&dbase=pubmed&querytype=eSearch&) | Search **((aorta[tiab] OR aortal[tiab] OR aortic[tiab] OR arteries[tiab] OR artery[tiab] OR arterial[tiab]) AND (stenosi*[tiab] OR thrombosi*[tiab])) OR "Peripheral Vascular Diseases"[MesH] OR "Arteries"[Mesh]** | 03:57:06 | [414086](http://www.ncbi.nlm.nih.gov/pubmed/?cmd=HistorySearch&querykey=8&) |
| [#7](http://www.ncbi.nlm.nih.gov/pubmed/?querykey=7&dbase=pubmed&querytype=eSearch&) | Search **#6 NOT #5** | 03:54:03 | [201](http://www.ncbi.nlm.nih.gov/pubmed/?cmd=HistorySearch&querykey=7&) |
| [#6](http://www.ncbi.nlm.nih.gov/pubmed/?querykey=6&dbase=pubmed&querytype=eSearch&) | Search **#1 AND #3 AND #4** | 03:53:43 | [319](http://www.ncbi.nlm.nih.gov/pubmed/?cmd=HistorySearch&querykey=6&) |
| [#5](http://www.ncbi.nlm.nih.gov/pubmed/?querykey=5&dbase=pubmed&querytype=eSearch&) | Search **#1 AND #2 AND #4** | 03:53:22 | [2355](http://www.ncbi.nlm.nih.gov/pubmed/?cmd=HistorySearch&querykey=5&) |
| [#4](http://www.ncbi.nlm.nih.gov/pubmed/?querykey=4&dbase=pubmed&querytype=eSearch&) | Search **(dutch[la] OR english[la] OR german[la]) NOT (animals[mesh] NOT humans[mesh])** | 03:52:59 | [13015971](http://www.ncbi.nlm.nih.gov/pubmed/?cmd=HistorySearch&querykey=4&) |
| [#3](http://www.ncbi.nlm.nih.gov/pubmed/?querykey=3&dbase=pubmed&querytype=eSearch&) | Search **((("Brain Infarction"[Mesh]) OR "Carotid Artery Thrombosis"[Mesh]) OR "Carotid Stenosis"[Mesh]) OR "Intracranial Embolism and Thrombosis"[Mesh] OR ((((cerebral[tiab] OR intracranial[tiab]) AND (artery[tiab] OR arteries[tiab] OR arterial[tiab])) OR brain[tiab]) AND infarct*[tiab]) OR (cerebrovascular[tiab] OR intracranial[tiab] OR brain[tiab]) AND (disorder*[tiab] OR disease*[tiab] OR insufficienc*[tiab] OR occlusion*[tiab]) OR (carotid [tiab] AND (disorder*[tiab] OR disease*[tiab]))** | 03:52:51 | [152157](http://www.ncbi.nlm.nih.gov/pubmed/?cmd=HistorySearch&querykey=3&) |
| [#2](http://www.ncbi.nlm.nih.gov/pubmed/?querykey=2&dbase=pubmed&querytype=eSearch&) | Search **((("Coronary Stenosis"[Mesh]) OR "Coronary Thrombosis"[Mesh]) OR "Arteriosclerosis"[Mesh]) OR (coronar*[tiab] AND (stenosi*[tiab] OR thrombosi*[tiab])) OR arteriosclerosi*[tiab] OR atherosclerosi*[tiab] OR (vascular[tiab] AND (disorder*[tiab] OR disease*[tiab])) OR (cardiovascular[tiab] AND (disorder*[tiab] OR disease*[tiab]))** | 03:52:11 | [308626](http://www.ncbi.nlm.nih.gov/pubmed/?cmd=HistorySearch&querykey=2&) |
| [#1](http://www.ncbi.nlm.nih.gov/pubmed/?querykey=1&dbase=pubmed&querytype=eSearch&) | Search **(("Osteoporosis"[Mesh:noexp] OR "Osteoporosis, Postmenopausal"[Mesh]) OR "Bone Density"[Mesh] OR osteoporos*[tiab] OR "bone loss"[tiab] OR "bone losses"[tiab] OR (bone[tiab] AND (densit*[tiab] OR content*[tiab])))** | 03:49:53 | [89159](http://www.ncbi.nlm.nih.gov/pubmed/?cmd=HistorySearch&querykey=1&) |
