# Supplementary material for: (Sub)clinical cardiovascular disease is associated with increased bone loss and fracture risk; a systematic review of the association between cardiovascular disease and osteoporosis
Source: Arthritis Res Ther. 2011 Jan 17;13(1):R5. doi: 10.1186/ar3224 (PMC3241350; doi:10.1186/ar3224)
Supplement: Additional file 2 — Quality assessment cohort studies. List of quality assessment of cohort studies as proposed by the Dutch Cochrane Collaboration. [file ar3224-S2.DOC]

Quality assessment cohort studies

1. Was the study population clear defined?
2. Was selection bias sufficiently accounted for?
3. Was the exposure clear defined and was the method appropriate?
4. Was the outcome clear defined and was the method appropriate?
5. Was the outcome blinded assessed? If the outcome was not blinded; did this influence the outcome?
6. Was the follow-up sufficiently long?
7. Was selective loss to follow-up appropriately prevented?
8. Are the most important confounders identified and is this adequately accounted for in the design and analyses?
9. Were the results valid and applicable? If not, the checklist could be stopped
10. Summary of the mean results
11. Are the results clinically relevant?

All items had the following answer options: yes/no/to little information to answer the question. We considered incomplete information/data an important criteria for study quality. Therefore, if the answer could not be given, because the study provided to little information, a negative score (e.g. “no”) was given. Each “no” was scored and an equal weight was given to each item. A maximum of 10 points can be given.
